# Gamma-Carbolines Derivatives As Promising Agents for the Development of Pathogenic Therapy for Proteinopathy

**Published:** 2018

**Authors:** V. I. Skvortsova, S. O. Bachurin, A. A. Ustyugov, M. S. Kukharsky, A. V. Deikin, V. L. Buchman, N. N. Ninkina

**Affiliations:** Pirogov Russian National Research Medical University, Ostrovitianov Str., 1, Moscow, 117997, Russia; Institute of Physiologically Active Compounds of the Russian Academy of Sciences, Severny Dr., Chernogolovka, 1142432, Russia; Institute of Gene Biology of the Russian Academy of Sciences, Vavilova Str., 34/5, Moscow, 119334 , Russia; Cardiff University, School of Biosciences, Sir Martin Evans Building, Museum Ave., Cardiff, CF10 3AX

**Keywords:** ALS, Dimebon, gamma-carbolines, proteinopathy, transgenic animals

## Abstract

Uncontrolled protein aggregation, accompanied by the formation of specific inclusions, is a major component of the pathogenesis of many common neurodegenerative diseases known as proteinopathies. The intermediate products of this aggregation are toxic to neurons and may be lethal. The development strategy of pathogenic therapy for proteinopathy is based on the design of drugs capable of both inhibiting proteinopathy progression and increasing the survival of affected neurons. The results of a decade-long research effort at leading Russian and international laboratories have demonstrated that Dimebon (Latrepirdine), as well as a number of its derivatives from a gamma-carboline group, show a strong neuroprotective effect and can modulate the course of a neurodegenerative process in both in vitro and in vivo model systems. The accumulated data indicate that gamma-carbolines are promising compounds for the development of pathogenic therapy for proteinopathies.

## INTRODUCTION

Uncontrolled aggregation of certain proteins, with the formation of histopathological inclusions (proteinopathy), is a major aspect of the pathogenesis of many neurodegenerative diseases (NDDs), including amyotrophic lateral sclerosis (ALS). Hence, the development of drugs capable of inhibiting proteinopathy progression is considered as an important direction in the development of pathogenic therapy for NDDs. Data from recent studies, which have been independently obtained at various laboratories in different countries, have convincingly proved the ability of a drug that belongs to a gamma-carboline group – Dimebon (Latrepirdine) – to effectively inhibit the progression of model proteinopathies in various transgenic animals. Our findings demonstrate the efficacy of Dimebon and its derivatives in inhibiting proteinopathy progression in model transgenic systems with a ALS phenotype.

Amyotrophic lateral sclerosis is a serious condition of the nervous system, with specific loss of motor neurons, and it is a type of proteinopathy caused by the aggregation of certain proteins. The association between pathogenic aggregation of these proteins and the development of a ALS phenotype has been demonstrated in numerous experimental studies on the modeling of the main mechanisms of a neurodegenerative process affecting motor neurons [1-3]. In a histopathological analysis of idiopathic ALS, the autopsy material of most patients contains intracellular protein inclusions. Of particular interest are deposits formed by TDP-43 and FUS DNA/RNA-binding proteins [4-6]. The direct mechanisms underlying the pathogenic aggregation of these proteins, leading to dysfunction and death of motor neurons, may be a specific trait of a given protein. There is little doubt that the process of pathogenic protein aggregation plays an important role in the pathogenesis of all ALS forms and might be an obvious target for therapeutic interventions.

## NEUROPROTECTIVE PROPERTIES OF GAMMA-CARBOLINES

Data from independent studies at several laboratories have demonstrated that compounds belonging to the gamma-carboline class are potential neuroprotective agents leading to reduced levels of pathogenic aggregation and/or activating the intracellular defense mechanisms of controlled degradation of aggregated proteins [7, 8]. Initial findings for these gamma-carbolines properties were obtained in Dimebon studies showing a correction of the cognitive function in patients with Alzheimer’s disease (AD), which is the most common neurodegenerative disease in the proteinopathy group [9, 10]. Furthermore, clinical trials conducted at several centers have revealed a positive effect from Dimebon on the cognitive function of patients with Huntington’s disease [11]. Yet, phase III clinical trials have indicated that Dimebon treatment is not considered effective compared to other developed drugs for the pathogenic therapy of AD [12], which is most likely due to the extremely high heterogeneity of nosological forms of Alzheimer’s disease. However, the mechanisms of action of the drug and its derivatives on proteinopathy progression have remained the object of intense research at several laboratories [13]. For example, the results of a recent meta-analysis revealed a positive effect from Dimebon on neuropsychiatric status indicators in AD patients [14] and provided an additional incentive to continue research in this direction. In addition, in a homogeneous model system of transgenic animals, Dimebon was shown to inhibit the development of tau proteinopathy, which is one of the key components of AD pathology [15]. Another key proteinopathy in the pathogenesis of AD is cerebral amyloidosis, which was also inhibited by Dimebon in TgCRND8 [16-18] and 3xTg-AD [19] mice, but not in a 5xFAD model characterized by a more aggressive course of amyloidosis [20]. These data served as grounds for expanding the range of research areas of gamma-carbolines effects on the progression of other proteinopathies that play an important role in the pathogenesis of neurodegenerative diseases.

## EFFECT OF GAMMA-CARBOLINES ON THE PROGRESSION OF PROTEINOPATHIES ASSOCIATED WITH THE SPECIFIC INVOLVEMENT OF MOTOR NEURONS

Chronic administration of Dimebon to a transgenic mouse model with the *pan*-neuronal expression of gamma-synuclein which reproduced the main features of ALS pathogenesis [21, 22] delayed the progression of proteinopathy [23, 24]. In this case, there was a significant decrease in the level of aggregated detergent-insoluble gamma-synuclein isoforms in affected areas of the nervous system in transgenic mice [25] and a decrease in gamma-synuclein-reactive inclusions in the affected spinal cord parts of the experimental animals [21, 22]. This effect was more pronounced if administration was begun at the pre-symptomatic stage, long before the first manifestations of the pathological process, according to both clinical symptoms and histological analysis. The same feature of Dimebon was observed in SOD^1G93A^ transgenic mice: if Dimebon was administered long before the expected age of manifestations of ALS phenotype-associated symptoms, then the onset of model disease symptoms occurred later, leading to an increased lifespan for the animals [26]. However, if Dimebon administration was started at an age closer to the expected onset of model disease symptoms, then the drug effects were much less pronounced [27]. We confirmed the ability of Dimebon and its derivatives to inhibit proteinopathy progression in a FUS^1-359^ transgenic mouse line [28, 29] which was recently generated and represents an adequate model of specific involvement of motor neurons with the ALS phenotype. In the nervous system of these mice, similarly to patients with FUS-associated forms of ALS, the histopathological analysis reveals an accumulation of aberrant FUS isoforms in characteristic cytoplasmic protein aggregates. Both Dimebon and its derivatives could modify, albeit with different efficacies, the progression of FUS proteinopathy in the nervous system of the FUS^1-359^ mice [30]. For example, the lifespan of model animals treated with Dimebon increased statistically significantly. Furthermore, transfer of the FUS^1-359^ mouse line from the C57Bl/6J genetic background, which was initially used in most studies in various laboratories, to the CD-1 genetic background did not affect the proteinopathy-inhibiting effect of gamma-carbolines and may not be explained by increased sensitivity of the C57Bl/6J line to gamma-carbolines [30]. In addition to an increased lifespan, the FUS^1-359^ mice treated with Dimebon or its derivative were characterized by a delayed onset of model disease symptoms with the development of a pronounced ALS phenotype if administration of the compounds was initiated at early latent stages of FUS proteinopathy [31]. However, the exact mechanism of Dimebon action remains unclear. The existing data from biochemical studies, as well as experiments on cell cultures and animals, suggest that Dimebon is a multitarget drug capable of affecting many intracellular processes and various pathogenic pathways in neurons and other cells affected by neurodegenerative changes [7].

Particularly, Dimebon can modulate the functioning of receptors and channels, change the kinetics of signaling enzymes [9, 32-35], as well as stabilize mitochondrial activity [36, 37]. But perhaps, the most significant property of Dimebon, which makes it a basic compound in the development of approaches for the treatment of proteinopathy, lies in its ability to inhibit the accumulation of cellular pathogenic protein aggregates.

## GAMMA-CARBOLINE-BASED INHIBITION OF ACCUMULATION OF PATHOHISTOLOGICAL PROTEIN INCLUSIONS IN NEURONAL CYTOPLASM

The ability of Dimebon to prevent an accumulation of pathogenic protein inclusions in neuronal bodies was first demonstrated in our joint research with M. Hasegawa’s and M. Goedert’s laboratories on cell cultures producing the aberrant and highly aggregating RNA-binding protein TDP-43 [38, 39]. The effect was confirmed using another cell model with the aggregation of the RNA-binding protein FUS. We demonstrated that addition of Dimebon and/or its derivatives to cultured human neuroblastoma cells with FUS proteinopathy reduces both the amount of insoluble protein forms in the cytoplasmic fraction and the amount of protein inclusions formed in the cytoplasm (unpublished data). Subsequent studies performed on various model proteinopathy systems confirmed these effects, and in our view they are associated with the activation of the autophagosome system in Dimebon-treated groups [16, 40-42].

## CONCLUSION

The results of a decade-long research effort conducted at leading Russian and international laboratories have demonstrated that compounds from the gamma-carboline series are indeed capable of suppressing the progression of certain types of proteinopathies and, as in the case of ALS models, slow down the development of the model phenotype of neurodegenerative processes in vivo. It is the modulation of aggregation of the proteins involved in proteinopathy mechanisms that is considered as the major element behind the concept of developing pathogenic therapy for neurodegenerative diseases [43]. At present, there is enough supporting evidence for considering Dimebon and its derivatives as promising compounds for the development of new therapeutic agents with improved pharmacokinetics and efficacy, which may be used as part of a complex pathogenic therapy for socially significant neurodegenerative diseases.

## Abbreviations

ALS: amyotrophic lateral sclerosis
FUS: fused in sarcoma
NDD: neurodegenerative disease
TDP-43: transactive response DNA binding protein 43 kDa
